# Sema3a as a Novel Therapeutic Option for High Glucose-Suppressed Osteogenic Differentiation in Diabetic Osteopathy

**DOI:** 10.3389/fendo.2019.00562

**Published:** 2019-08-20

**Authors:** Lixia Zhang, Lili Zheng, Chong Li, Zhifang Wang, Shan Li, Lijun Xu

**Affiliations:** Department of Endocrinology, The First Affiliated Hospital of Zhengzhou University, Zhengzhou, China

**Keywords:** sema3a, therapy-, osteogenic differentiation, diabetes, osteopathy

## Abstract

**Objective:** Diabetic osteopathy is a common comorbidity of diabetes mellitus, with skeletal fragility, osteoporosis and bone pain. The aim of our study was to highlight the role of sema3a on osteoblast differentiation of MC3T3-e1 in high-glucose condition and explore its therapeutic effect of diabetic osteopathy *in vitro* and *vivo*.

**Methods:** In our study, the expression of osteogenesis-related makers, such as ALP, OCN, OPG, β-catenin and Runx2, were analyzed in MC3T3 osteoblastic cells to explore the effect of sema3a on osteoblast differentiation in high-glucose condition, and as was the staining of ALP and Alizarin Red S. In a diabetic animal model, the expression of serum bone metabolic markers, such as ALP, P1NP, OCN, and β-CTX, were analyzed and micro-CT was used to detect bone architecture, including Tb.N, Tb.Th, Tb.Sp, Tb.Pf, BS/BV, and BV/TV after the treatment of sema3a.

**Results:** High glucose significantly inhibited osteogenic differentiation by decreasing the expression of osteogenesis-related makers, sema3a and its receptor of Nrp-1 in a dose-dependent manner in MC3T3. In high-glucose condition, exogenous sema3a (RPL917Mu01) increased the expression of ALP, OCN, OPG, Runx2, β-catenin, and the positive proportion of ALP and Alizarin Red S staining. In addition, in diabetic animal model, exogenous sema3a could increase bone mass and bone mineral density, and downregulate the expression of ALP, P1NP, OCN, and β-CTX.

**Conclusion:** High glucose suppresses osteogenic differentiation in MC3T3 and sema3a may take part in this process. The application of exogenous sema3a alleviates high glucose-induced inhibition of osteoblast differentiation in diabetic osteopathy.

## Introduction

Diabetes, characterized by high blood glucose levels, is a worldwide chronic metabolic disease due to damages in insulin secretion or/and activity (1). It may also occur along with a series of complications due to durable hyperglycemia, such as periodontal disease, microvascular disease, nephropathy, retinopathy, neuropathy and osteopathy (2, 3). With regard to diabetic osteopathy, it often results in skeletal fragility, osteoporosis and bone pain (4). It not only decreases the life quality of patients, but also increases the social economic burdens. Thus, there is a pressing need to explore the pathogenic mechanism and find a new therapeutic strategy for diabetic osteopathy.

Several mechanisms have been explained to the imbalance homeostasis in diabetic osteopathy, such as glucose metabolism, inflammatory molecules, growth factors and bone turnover (3, 5, 6). In particular, as a key pathogenic mechanism, disturbed bone turnover contributes to the impaired bone formation, remodeling and decreased bone thickness (5). Therefore, targets for regulation of the balanced bone turnover are important for the treatment of diabetic osteopathy.

Semaphorin 3A (Sema3a), an important member from the large semaphorin family, is a well-known factor in the tumorigenesis and nervous, immune systems and bone development (7–10). During bone development, both osteoblasts and osteoclasts can express semaphorin family proteins, which can act as paracrine signaling molecules to regulate bone remodeling (11, 12). Sema3a, produced by osteoblasts, also has an osteoprotective effect. This process is usually activated by binding with Neuropilin 1 (Nrp-1), the cell-surface receptor of sema3a (13). Additionally, this binding not only activates canonical Wnt/β-catenin-related osteoblast differentiation, but also suppresses receptor activator of nuclear factor kappa beta ligand (RANKL)-related osteoclast differentiation (12, 14). Moreover, sema3a has been demonstrated to be a target to treat osteoporosis and the recombinant sema3a protein administration decreases bone loss in an ovariectomized mouse model (12).

However, the effect of sema3a on osteoblast differentiation in diabetic osteopathy is still unknown. Therefore, in this study, we aim to highlight the effect of high-glucose on osteoblast differentiation of MC3T3-e1 and explore the role of sema3a in this process. Moreover, we also explore the therapeutic effect of sema3a in diabetic osteopathy.

## Materials and Methods

### Cell Cultures

The osteoblastic MC3T3 cells were obtained from Cell Bank, Chinese Academy of Sciences (China) and were cultured in DMEM (Gibco, USA) at 37°C. The MC3T3cells were treated at high, medium and standard glucose DMEM (glucose concentration 25.0, 15.25, and 5.5 mmol/L) and the medium contained 10% fetal bovine serum (FBS) (Gibco), penicillin (100 U/ml), and streptomycin (100 mg/ml) (15). In addition, 5 mM β-glycerophosphate, dexamethasone and ascorbic acid (50 mg/ml) were used in culture medium to initiate differentiation.

### Alkaline Phosphatase (ALP) Assay

MC3T3 cells were seeded onto 24-wells plates with the density of 1 × 10^4^ cells/well. The period of culture medium renewal was 2 days. At the third and fifth day, cells were harvested, and then ALP staining (Sigma) was performed according to the manufacturer's protocol and observed via microscope (Nikon).

### Alizarin Red S Staining Assay

MC3T3 cells were seeded onto 12-well-plates with the density of 4 × 10^4^ cells/well and the period of culture medium renewal was 2 days. At the 14 and 21th day, cells were harvested, and fixed with 4% paraformaldehyde for 30 min at room temperature. After being washed three times with phosphate buffer saline (PBS), Alizarin Red S (Solarbio, China) was performed according to the manufacturer's protocol. Calcium nodules were imaged and counted via microscope (Nikon).

### Quantitative Real-Time PCR (qRT-PCR)

Total RNA was extracted from cells after maintaining the culture for 24, 36 and 72 h using TRIzol reagent (Gibco) as described previously (6). Sema3a, Nrp-1, OCN, OPG, ALP, β-catenin, and Runx2 were quantified on Mx 3005P (STRATAGENE) using DyNamo SYBR1 Green qPCR kit (Takara) and normalized with GADPH. The primer of the target gene was presented in Table 1.

**Table 1:** Primers sequence used for qRT-PCR analysis.

**Gene**	**Gene ID**	**Forward primer sequence (5′-3′)**	**Reverse primer sequence (5′-3′)**
Sema3a	NM_009152	CCCAAGGAGACTTGGCATG	CCCGCAGTTGAGCCAATG
Nrp-1	NM_008737	CAAGAGAGGGCCCGAATG	TGTAGGTGCACTCCAAGCTG
OCN	NM_007541	CTGCGCTCTGTCTCTCTGAC	TTAAGCTCACACTGCTCCCG
OPG	NM_008764	AGTGTGAGGAAGGGCGTTAC	AATGTGCTGCAGTTCGTGTG
ALP	NM_007431	CCGGCTGGAGATGGACAAAT	CTCATTGCCCTGAGTGGTG
β-catenin	NM_001165902	TGTGCAGCTGCTTTATTCTCCCA	TATGTCGCCACACCTTCATTCCTA
Runx2	NM_001146038	AAATGCCTCCGCTGTTATGAA	GCTCCGGCCCACAAATCT
GAPDH	NM_008084	AGGTCGGTGTGAACGGATTTG	GGGGTCGTTGATGGCAACA

*Sema3a, Semaphorin 3A; Nrp-1, Neuropilin 1; OCN, Osteocalcin; ALP, Alkaline phosphatase; OPG, Osteoprotegerin, Runx2, runt-related transcription factor 2*.

### Western Blot

Samples were resolved on SDS-PAGE gel. Proteins were transferred onto a PVDF membrane (Millipore, Germany) by conventional methods. It was probed with primary antibodies against sema3a (1:1,000, Abcam), Nrp-1 (1:1,000, CST), GAPDH (1:10,000, Abcam), and then the immunoreactive protein was visualized by using BeyoECL Plus kit (Beyotime, China).

### Flow Cytometry Analysis

Cell apoptosis analysis was performed based on a previous study (16). Cells cultured at high, medium and standard glucose DMEM were washed by PBS and stained successively by propidium iodide (PI) and fluorescein isothiocynate (FITC)-conjugated annexin V (FITC-annexin V). The apoptotic cells were sorted by FACScan (Beckman Coulter, Fullerton, CA) and analyzed by FlowJo software (Tree Star, San Carlos, CA).

### Sema3a Treatment *in vitro*

The MC3T3 cells were cultured at high, medium and standard glucose DMEM and treated with exogenous seme3a (RPL917Mu01, wuhan UCSN Business Co., Ltd, China) with the dose of 100 and 500 ng/ml, respectively.

### Animal Treatments and Sample Preparation

Eight-week-old male C57BL/6 mice were purchased from Animal Experimental Center, Guangdong Academy of Medical Sciences (Guangzhou, China). This study was approved by the Life Science Ethics Review Committee of Zhengzhou University. According to the protocol of our Local Committee for Animal Care and Ethics, a total of 24 mice were divided into the following groups randomly: non-diabetes (*n* = 8), non-treatment diabetes (*n* = 8) and sema3a-treatment diabetes (*n* = 8). All the mice were raised in a temperature- and light-controlled environment of 26–28°C and equal light-darkness-cycle. Additionally, they were fed standard laboratory fodder and water *ad libitum*.

Diabetes was induced by streptozotocin (STZ, Sigma S0130), which was freshly dissolved in the sodium citrate buffer in two experimental groups. The diabetogenic agent was injected intraperitoneally at a dose of 150 mg/kg, while control group of eight mice were simulated this intraperitoneal injection with an equivalent volume of sodium citrate buffer (17, 18). Mice in the group of non-treatment diabetes and sema3a-treatment diabetes received protamine zinc insulin (Humulin-N, Eli Lilly and Company) intraperitoneally to control the blood glucose. Subsequently, sema3a (recombinant semaphorin 3A, HZbscience) was injected intraperitoneally at a dose of 100 ng/g body weight in sema3a-treatment diabetes mice and the isovolumetric DMSO was injected intraperitoneally in non-treatment diabetes mice (19, 20). Blood glucose and weight was measured and recorded at fixed period (0, 2, 4, 6, 8, 10, 12 weeks).

### Assay of ALP, P1NP, OCN, and CTX in Serum

Non-fasting blood samples were obtained from all mice before sacrifice and stored at −20°C to evaluate the expression of serum bone biochemical markers. The serum bone biochemical markers were detected with the method of enzyme-linked immunosorbent assay (ELISA) kit. The ELISA kit of ALP, procollagen type 1 amino terminal propeptide (P1NP), C-terminal cross-linked telopeptide of type-I collagen (CTX) and osteocalcin (OCN) were provided by Nanjing Jiancheng Bioengineering Institute (China), with osteocalcin (OCN) by R&D Systems (USA) and HbA1C by Hangzhou Jianglai Biotechnology Co. (China).

### Micro CT Analysis

After 12 weeks, all mice were executed by rapid cervical dislocation under anesthesia. Right femurs were obtained and wipe off soft tissue. Following dissection, the obtained femurs were scanned using a GE eXplore Locus SP Micro-CT imaging system (GE Medical Systems, Canada). Three-dimensional images of the targeted femurs were reconstructed. Trabecular bone microarchitecture, such as the trabecular number (Tb.N) [/mm], trabecular thickness (Tb.Th) [μm], trabecular spacing (Tb.Sp) [μm], trabecular pattern factor (Tb.Pf), Bone Surface Area/Bone Volume (BS/BV) [%] and the indexes of bone volume per total volume (BV/TV) [%] were analyzed within the targeted zone (21).

### Statistical Analysis

All results have been obtained from at least three independent experiments and the experimental data presented as the mean ± standard deviations (S.E.). Statistical significance among different experimental groups was evaluated by one-way analysis of variance (ANOVA) and two-way ANOVA using SPSS 17.0 software and significance was considered as ^*^p < 0.05 and ^**^p < 0.01.

## Results

### Effect of High Glucose on the Osteogenic Differentiation of MC3T3

**Figure 1:**
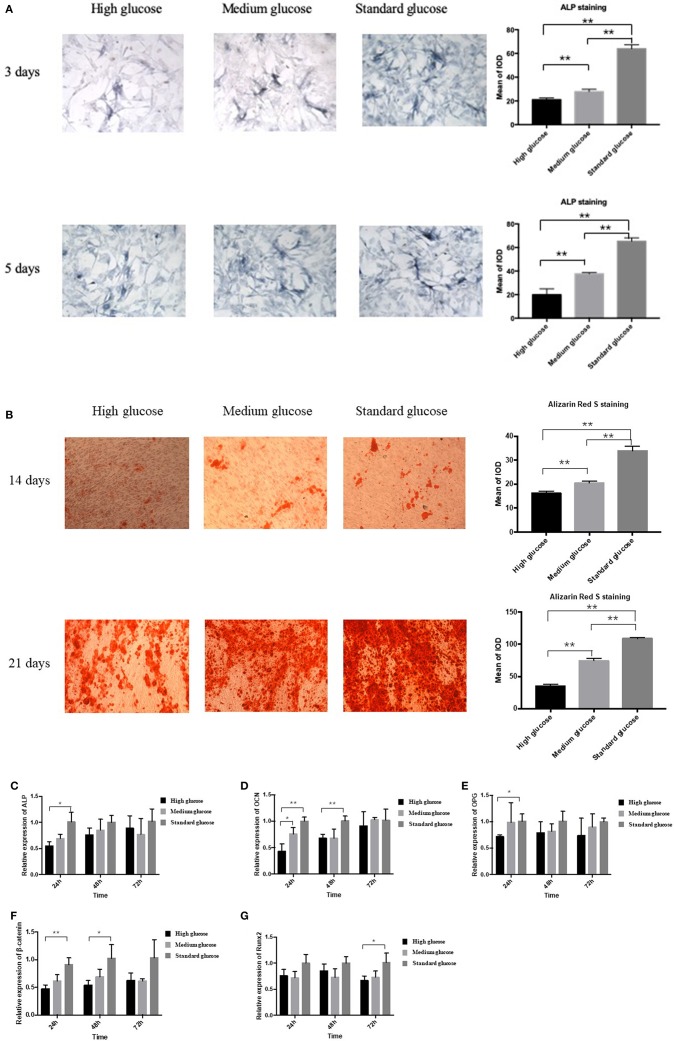
Effect of high glucose on the osteogenic differentiation of MC3T3. **(A)** The ALP staining of MC3T3 cultured in different medium (high, medium and standard glucose DMEM) at day 3 and 5, and quantitative analysis; **(B)** The Alizarin Red S staining of MC3T3 cultured in different medium (high, medium and standard glucose DMEM) at day 14 and 21, and quantitative analysis; The mRNA expression of ALP **(C)**, OCN **(D)**, OPG **(E)**, β-catenin **(F)** and Runx2 **(G)** of MC3T3 cultured in different medium (high, medium and standard glucose DMEM) at 24, 48, and 72 h. The glucose concentration of high, medium and standard glucose DMEM is 25.0, 15.25, and 5.5 mmol/L, respectively. Columns show the mean results of experiments carried out with triplicate; bar = SD. ^*^P < 0.05, ^**^P < 0.01.

In order to explore the effect of high glucose on the osteogenesis of MC3T3 cells, ALP staining was performed. High and medium glucose groups resulted in reduction of osteogenesis at both day 3 and 5 significantly (*p* < 0.01, Figure 1A). Alizarin Red S staining also demonstrated that high-glucose could significantly decrease the Alizarin Red S-calcium complex at both day 14 and 21 (*p* < 0.01, Figure 1B). In addition, no significant difference of apoptosis cells was found among three groups (Supplementary Figure 1).

In the two-way ANOVA, compared with standard glucose, high glucose significantly decreased the expression of the osteogenesis-related makers of ALP, osteoprotegerin (OPG), β-catenin and OCN at 24 h, decreased the expression of the OCN and β-catenin at 48 h and the runt-related transcription factor 2 (Runx2) at 72 h (Figures 1C–G). Additionally, medium glucose also decreased the expression of OCN at 24 h (Figure 1D).

### The Expression of Sema3a and Its Receptor of Nrp-1 in High-Glucose Condition in MC3T3

**Figure 2:**
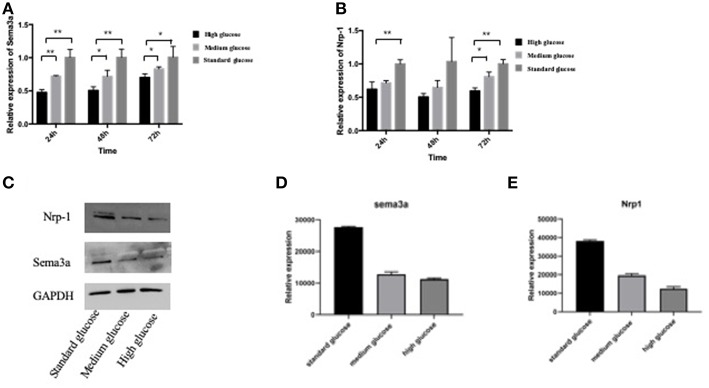
The mRNA expression of sema3a **(A)** and Nrp-1 **(B)** of MC3T3 cultured in different medium at 24, 48, and 72 h. **(C)** The Western Blot of sema3a and Nrp-1. The quantification analysis of the Western Blot of sema3a **(D)** and Nrp-1 **(E)**. The medium is high, medium and standard glucose DMEM, and the glucose concentration is 25.0, 15.25, and 5.5 mmol/L, respectively. Columns show the mean results of experiments carried out with triplicate; bar = SD. ^*^P < 0.05, ^**^P < 0.01.

In order to explore the expressions of sema3a and its receptor of Nrp-1 in different glucose concentration medium, MC3T3 cells were cultured in high, medium and standard glucose DMEM. High glucose caused a significant decrease of sema3a and Nrp-1 in a dose-dependent manner (Figures 2A,B). The mRNA expression of sema3a at 72 h was significantly higher than that at 24 and 48 h (*p* < 0.05), while the mRNA expression of Nrp-1 was not significant different in a time-dependent manner (Figures 2A,B). In addition, the protein expression of sema3a and Nrp-1 also decreased with the treatment of high glucose (Figures 2C–E).

### The Effects of Exogenous Sema3a on High-Glucose-Induced Osteogenic Differentiation *in vitro*

**Figure 3:**
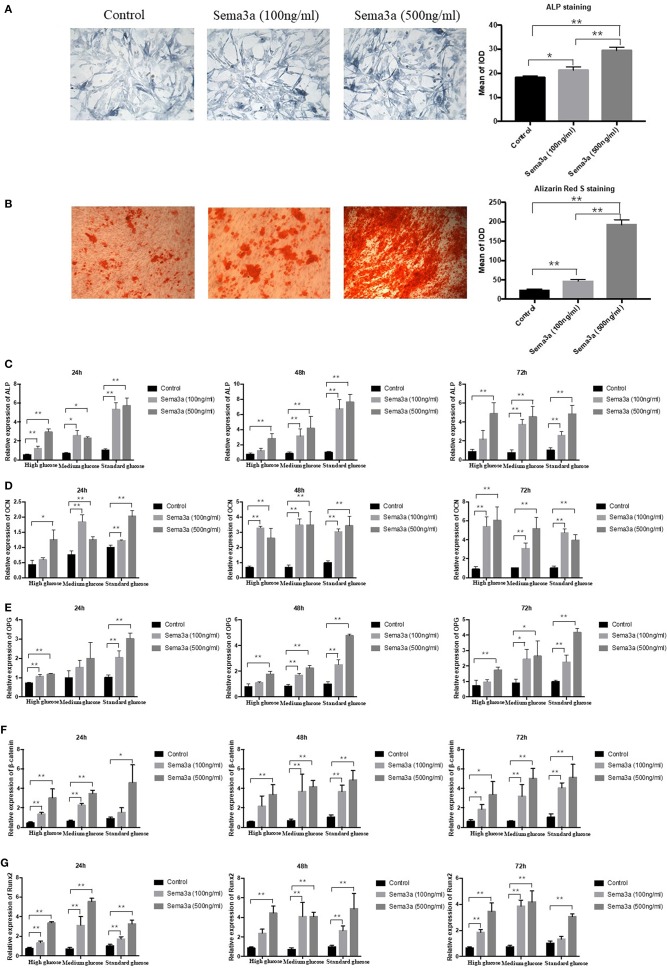
The effects of exogenous sema3a on high-glucose-induced osteogenic differentiation *in vitro*: **(A)** The ALP staining of MC3T3 cultured in high-glucose DMEM treated with different doses of sema3a at day 5, and quantitative analysis; **(B)** The Alizarin Red S staining of MC3T3 cultured in high-glucose DMEM treated with different doses of sema3a at day 21, and quantitative analysis; The mRNA expression of ALP **(C)**, OCN **(D)**, OPG **(E)**, β-catenin **(F)**, and Runx2 **(G)** of MC3T3 cultured in different medium (high, medium and standard glucose DMEM) treated with different doses of sema3a at 24, 48, and 72 h. Columns show the mean results of experiments carried out with triplicate; bar = SD. ^*^P < 0.05, ^**^P < 0.01.

In order to further explore the effect of sema3a on high glucose-suppressed osteogenic differentiation, cells cultured in high glucose DMEM were treated with different dose of exogenous sema3a. ALP and Alizarin Red S staining were used to evaluate the osteogenesis process. ALP staining showed that exogenous sema3a (100 and 500 ng/ml) resulted in the increase of ALP production at day 5 (Figure 3A). Also, calcium nodule deposition of Alizarin Red S staining in treatment groups increased obviously at day 21. Additionally, the increase presented in a dose-dependent manner (Figure 3B). Therefore, exogenous sema3a recovered osteogenic differentiation destroyed by high glucose.

Subsequently, the expression of ALP, OCN, OPG, β-catenin, and Runx2 mRNA was analyzed by qRT-PCR at 24, 48, and 72 h. As presented in Figures 3C–G, the two-way ANOVA revealed that the expression of ALP, OPG, β-catenin, OCN, and Runx2 was highest with the treatment of exogenous sema3a (500 ng/ml) and that was lowest in the control group, which demonstrated that exogenous sema3a recovered high glucose-caused suppression of ALP, OCN, OPG, β-catenin, and Runx2 mRNA expression in a dose-dependent manner. In the one-way ANOVA, the expression levels of ALP, OCN, OPG, β-catenin, and Runx2 were significantly increased in all groups with the treatment of sema3a (500 ng/ml) at 24, 48, and 72 h, except OPG of medium glucose group at 24 h. The dose of 100 ng/ml also significantly upregulated their expression in most groups except ALP of high glucose group at 48 and 72 h, OCN of high glucose group at 24 h, OPG of medium glucose group at 24 h, OPG of high glucose group at 48 and 72 h, β-catenin of high glucose group at 48 h and standard glucose group at 24 h, Runx2 of high glucose group at 48 h and standard glucose group at 72 h.

### The Effects of Exogenous Sema3a on High-Glucose-Induced Osteogenic Differentiation *in vivo*

**Figure 4:**
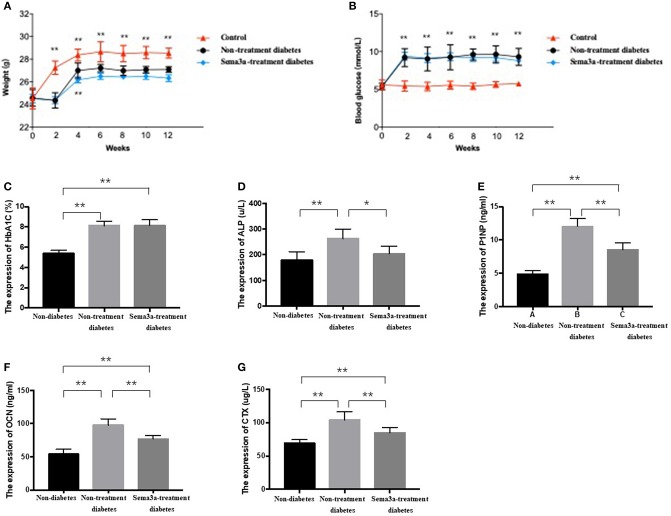
Effects of exogenous sema3a on **(A)** body weight and **(B)** blood glucose in each experimental group at 0, 2, 4, 6, 8, 10, 12 weeks; The serum biomarkers of HbA1c **(C)**, ALP **(D)**, P1NP **(E)**, OCN **(F)**, and β-CTX **(G)** in each experimental group at 12 weeks. The group is non-diabetic mice, non-treatment diabetic mice, and sema3a-treatment diabetic mice, respectively. Values are presented as means ± SD. ^*^P < 0.05, ^**^P < 0.01.

Glucose metabolism and body weight were used to evaluate the mice within 12 weeks. The mean weight of non-treatment diabetic mice was 26.33 g and sema3a-treatment diabetic mice was 25.83 g. The weight of both groups was significantly lower than that of non-diabetic mice from 2 weeks to 12 weeks (*p* < 0.01) (Figure 4A). Although the mean weight of non-treatment diabetic mice was higher than that of sema3a-treatment diabetic mice from 4 to 12 weeks, the significant difference was only found at 4 weeks (Figure 4A). Non-fasting plasma glucose of diabetic mice was significantly higher than that of non-diabetic mice from 2 to 12 weeks (*p* < 0.01), with the mean plasma glucose of 5.53 mmol/L for non-diabetic mice, 8.76 mmol/L for non-treatment diabetic mice and 8.63 mmol/L for sema3a-treatment diabetic mice (Figure 4B). The serum HbA1c was increased significantly in non-treatment diabetic mice and sema3a-treatment diabetic mice (*p* < 0.01, Figure 4C). The detailed information was listed in Tables 2, 3.

**Table 2:** The glucose and body weight in each experimental group.

**Time (weeks)**		**Glucose (mg/dL)**			**Body weight(g)**	
	**Control**	**Diabetes**	**Diabetes-sema3a**	**Control**	**Diabetes**	**Diabetes-sema3a**
0	5.6 ± 0.64	5.37 ± 0.44	5.41 ± 0.44	24.52 ± 0.92	24.58 ± 0.74	24.51 ± 1.07
2	5.42 ± 0.68	9.19 ± 1.19	9.38 ± 1.45	27.24 ± 0.59	24.36 ± 0.68	24.38 ± 0.86
4	5.36 ± 0.57	9.01 ± 1.58	9.13 ± 1.68	28.36 ± 0.53	27 ± 0.67	26.17 ± 0.74
6	5.54 ± 0.52	9.25 ± 1.67	9.31 ± 1.39	28.66 ± 0.87	27.22 ± 0.33	26.49 ± 0.72
8	5.4 ± 0.43	9.60 ± 0.74	9.2 ± 1.32	28.5 ± 0.71	26.98 ± 0.42	26.43 ± 0.59
10	5.63 ± 0.37	9.62 ± 1.14	9.2 ± 1.19	28.58 ± 0.55	27.07 ± 0.29	26.49 ± 0.66
12	5.75 ± 0.16	9.29 ± 1.13	8.8 ± 0.41	28.53 ± 0.43	27.09 ± 0.25	26.32 ± 0.73

**Table 3:** The serum bone turnover markers in each experimental group.

**Parameter**	**Control**	**Diabetes**	**Diabetes-sema3a**
HbA1C (%)	5.38 ± 0.31	8.13 ± 0.44	9.14 ± 0.57
ALP (u/L)	178.06 ± 34.12	263.07 ± 37.62	203.45 ± 30.17
P1NP (ng/ml)	4.89 ± 0.5	12.02 ± 1.19	8.12 ± 3.17
OCN (ng/ml)	53.89 ± 7.41	97.43 ± 9.65	76.79 ± 5.25
CTX (ug/L)	69.29 ± 5.18	104.11 ± 12.17	84.94 ± 7.99

*ALP, Alkaline phosphatase; P1NP, Procollagen Type 1 Amino Terminal Propeptide; OCN, Osteocalcin; CTX, C-terminal cross-linked telopeptide of type-I collagen*.

The expression of bone metabolic markers, including ALP, P1NP, OCN, and β-CTX, were detected by ELISA Kit in different groups. As shown in Figures 4D–G, compared with non-diabetic mice and sema3a-treated diabetic mice, the serum ALP, P1NP, OCN, and β-CTX was significantly upregulated in non-treatment diabetic mice. Compared with non-diabetic mice, the serum P1NP, OCN, and β-CTX was significantly increased in sema3a-treated diabetic mice. The mice in sema3a-treated diabetic mice showed a slightly higher level of ALP than non-diabetic mice. The detailed information was listed in Table 3.

**Figure 5:**
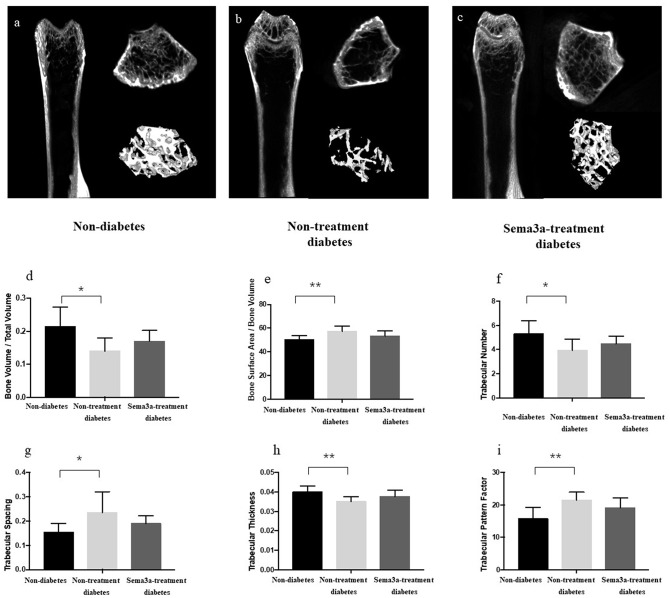
Effects of sema3a treatment on micro-CT and structural bone parameters. The coronal, axial and three-dimensional reconstruction micro-CT images of mice femur in non-diabetic mice **(a)**, non-treatment diabetic mice **(b)**, and sema3a-treatment diabetic mice **(c)** group; **(d)** bone volume per total volume (BV/TV); **(e)** bone Surface Area/Bone Volume (BS/BV); **(f)** trabecular number (Tb.N); **(g)** trabecular spacing (Tb.Sp); **(h)** trabecular thickness (Tb.Th) and **(i)** trabecular pattern factor (Tb.Pf), were also examined in femur. Values are presented as means ± SD. ^*^P < 0.05, ^**^P < 0.01.

Micro-CT was used to detect the bone mass of femur and showed ascoronal, axial, and three-dimensional reconstruction images. The bone mass of non-diabetic mice was higher than the other two groups and the treatment of sema3a increase the bone mass compare with non-treatment diabetic mice (Figures 5a–c). As shown in Figures 5d–i, when compared with non-diabetic mice, the value of BV/TV, Tb.N and Tb.Th in non-treatment diabetic mice reduced significantly (*P* < 0.05), while Tb.Sp, BS/BV, and Tb.Pf increased in non-treatment diabetic mice (*P* < 0.05), which demonstrated that the bone mass of diabetic mice was lower than that of non-diabetic mice. All of the above-mentioned factors were not significantly different between non-diabetic mice and sema3a-treatment diabetic mice, which demonstrated that exogenous sema3a recovered high glucose-bone mass loss.

## Discussion

Diabetic osteopathy, a widely-recognized complications of diabetes, often results in skeletal fragility, osteoporosis and bone pain due to the effect of high glucose on osteogenic differentiation. However, its molecular mechanism is not completely understood. Thus, in this study, we chose different glucose concentrations to present standard-, medium- and high-glucose condition similar to those in diabetic. In our present finding, we observed that high glucose suppressed osteogenic differentiation by targeting sema3a, which suggest that exogenous sema3a may serve as a method to recover the high glucose-suppressed osteogenic differentiation. Then we detected the role of exogenous sema3a on osteogenic differentiation in high-glucose condition and, as expected, our results proved the above-mentioned hypothesis *in vitro* and *in vivo*.

Hyperglycemia is the most pathological features of diabetic osteopathy, and is thought to be the cause of imbalanced bone turnover (3). Bone turnover requires the coupling of osteoblasts-associated bone formation and osteoclasts-associated bone resorption (22). Various coupling factors take part in this process and maintain bone remodeling homeostasis (23). These bone-related markers can be divided into two parts, that is bone formation and bone resorption markers. The former consists of ALP, OCN, OPG, and P1NP (24, 25), while the latter consists of CTX, tartrate-resistant acid phosphatase (TRAP), RANKL and sclerostin (Scl) (26). Runx2 is an important regulator in osteoblast differentiation and can also activate the expression of other osteogenic-related genes (27). In our present finding, we observed that high glucose suppressed osteogenic differentiation in MC3T3 cells in a dose- and time-dependent manner by ALP and Alizarin Red S staining and those bone formation markers, which were used to estimate osteogenic differentiation.

In addition, we also found that high glucose suppressed osteogenic differentiation by targeting sema3a and its receptor of Nrp-1. Nowadays, sema3a is suggested to work in the development of diabetes, diabetic complication and osteoporosis (28). In diabetic nephropathy, sema3a was regarded as a target of miR-15b-5p to induce podocyte injury (29). Moreover, its plasma level was correlated with the phenotypes of diabetic retinopathy and albuminuria, which suggested that it could be a potential biomarker (30). Sema3a, secreted by osteoblastic cells, is a dual function coupling factor to inhibit osteoclastic activity and promote osteoblastic differentiation (12). It has been found that mice lacking sema3a underwent bone loss (14). Therefore, regulation of sema3a may be helpful for preventing bone loss (31). In order to demonstrate this hypothesis in diabetic osteopathy, we next explored the effect of exogenous sema3a on high glucose-suppressed osteogenic differentiation. We observed that the bone formation biomarkers increased significantly after the treatment of exogenous sema3a. Thus, sema3a is thought to recover high glucose-suppressed osteogenic differentiation in a dose- and time-dependent manner *in vitro*.

As we all know, bone turnover and metabolism could not be fully reflected by *in vitro* tests (32). Thus, we constructed diabetic animal model and tested the effect of exogenous sema3a in diabetic osteopathy. Non-fasting plasma glucose of diabetic mice was higher than that of non-diabetic mice significantly, as well as HbA1c. With regard to body weight of diabetic mice, it was lower than that of non-diabetic mice, similar to previous studies (33, 34). Thus, the diabetic animal model was constructed successfully. In the meantime, we found that exogenous sema3a did not affect glucose metabolism and body weight in diabetic osteopathy. It revealed that sema3a was not a therapeutic method for diabetes.

In diabetic mice, both bone formation (ALP, OCN, PINP) and bone resorption (CTX-1) markers were increased compared to in non-diabetic animals, indicating an increased bone turnover. Similar to previous studies, CTX-1, the serum bone resorption marker, was increased in diabetic mice, which may suggest enhanced osteoclast differentiation (35). Interesting, different from bone formation biomarkers *in vitro*, the expression of ALP, osteocalcin, and PINP in the serum were increased in diabetic mice, which can be explained by the theory that osteoblasts was stimulated to compensate for the insulin depletion-related bone loss (33). The same situation of increased ALP is also found in diabetes patients, which is explained by the matrix hypermineralization (22, 23). After the treatment of sema3a, although the bone metabolic markers were still higher than those in non-diabetic mice, the difference is not significant. Thus, the application of exogenous sema3a recovered the changes of serum bone metabolic markers.

The bone mass of femur was detected by micro-CT and after evaluation, the value of BV/TV, Tb.N and Tb.Th reduced significantly, while Tb.Sp, BS/BV, and Tb.Pf increased in non-treatment diabetic mice, which demonstrated that in diabetic mice, the bone mass and bone mineral density were lower than those in non-diabetic mice. All the above-mentioned factors were not significantly different between non-diabetic rats and sema3a-treatment diabetic rats, which proved that exogenous sema3a recovered high glucose-bone mass loss.

## Conclusion

In conclusion, our data indicate that high glucose suppresses osteogenic differentiation in MC3T3 and sema3a may take part in this process. Therapeutically, the application of exogenous sema3a recovers high glucose-suppressed osteogenic differentiation *in vitro* and *in vivo*.

## Data Availability

Data will be made available on request.

## Author Contributions

All authors listed have made a substantial, direct and intellectual contribution to the work, and approved it for publication.

### Conflict of Interest Statement

The authors declare that the research was conducted in the absence of any commercial or financial relationships that could be construed as a potential conflict of interest.

## Supplementary Material

Supplementary Figure 1 Effect of high glucose on the cell apoptosis of MC3T3 by flow cytometry.
