# Supplementary figures and images for: Sema3a as a Novel Therapeutic Option for High Glucose-Suppressed Osteogenic Differentiation in Diabetic Osteopathy
Source: Front Endocrinol (Lausanne). 2019 Aug 20;10:562. doi: 10.3389/fendo.2019.00562 (PMC6710340; doi:10.3389/fendo.2019.00562)

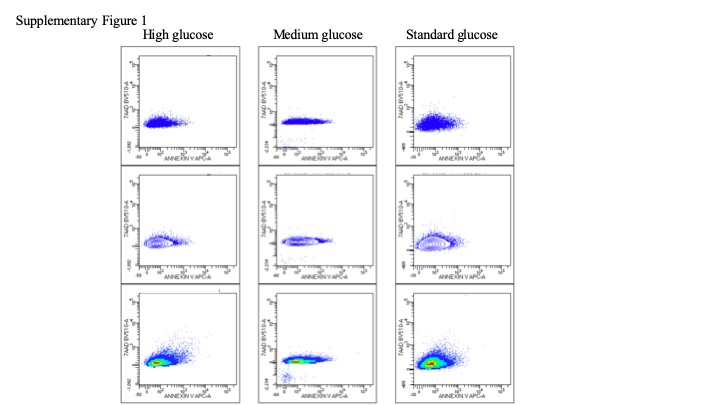

Supplement: Supplementary Figure 1 — Effect of high glucose on the cell apoptosis of MC3T3 by flow cytometry. [file Image_1.JPEG]
